# The Pneumonia Mortality

**Published:** 1919-04-26

**Authors:** 


### THE PNEUMONIA MORTALITY.

### With Special Reference to the Influenza Complication.

Before any of the recent outbreaks had begun to play havoc with the populations of practically every quarter of the globe, we were often told that pneumonia was the cause of more deaths than any other disease. To-day, in spite of an army of newly virulent infections and the widespread violence of war, this statement is more true than ever, but of late both the clinical picture and the post-mortem findings in lung complications differed markedly from the classical types of pre-war days. Requirements of treatment have correspondingly changed, and the very facts of the relative failure of all our older methods to combat successfully the extension of "influenzal" infections established either in the lung substance or the blood stream, emphasise very strongly our need to revise our customary procedures.

It is not our intention to dissertate on the diagnosis and treatment of pneumonia in a general way, such material relating to the classical disease can be found in any text-book. Rather would we make a brief review of some observations made in the recent literature of both this country and America.

### Types of Lung Infection.

Speaking generally, it is only in the mild cases that instances of true lobar pneumonia have been seen. In practically all those which, from the foot of the bed, one would describe as severe—they number about 20 per cent. of the whole—although lung signs and symptoms have been predominant, yet there has been hardly any organ in the body unaffected. In fact, there seems little doubt that the terms generalised toxæmia or actual septicæmia are far more descriptive of the condition than the perhaps more convenient label pneumonia. Certainly in a great many cases there has been dulness over a whole lobe of the lung with most of the typical "lobar" signs, but, when examined post-mortem, such a lobe has rarely shown the usual red or grey hepatisation. Instead an extraordinary complexity of lesions is observed, sometimes confined to one lung, more often scattered over both, and exhibiting totally distinct pathological conditions in the various areas involved. Broncho-pneumonia, œdema, congestion, hæmorrhage in the lung substance, areas of collapse, abscesses, single or multiple, infarcts, interstitial emphysema, bronchitis, or even gangrenous patches may be found in a series of variable combinations. The actual lesions are widely different from typical croupous pneumonia in both character and distribution. Such are the views of Dr. Herbert French, who has had great experience of the disease in the Army of this country, and from whose recent lectures many of our points are taken.

*X*-ray appearances, extensively studied in the United States, bear out this unusual development. Relatively few give a typical, persistent lobar shadow. The common discreet, scattered broncho-pneumonic type sometimes observed gave a normal percentage recovery, but of the severe cases, many of which died within one week, the usual condition demonstrated in life by *x*-rays and confirmed post-mortem exhibited, to quote the American authors' words, streptococcal and massive broncho-pneumonia with multiple abcesses, gangrene, pulmonary œdema and fibrino-purulent pleurisy with effusion, tracheitis and bronchitis.

### Prognostic Indications.

During the influenza outbreaks, about twenty per cent. of cases belonged to this severe class, about fourteen per cent. came to the point of death, and eight per cent., in spite of any possible treatment, had a rapidly fatal ending. The pulse-rate of the men was of surprisingly poor prognostic assistance, and in those obviously certain to die it was frequently less than 100 until the last few hours of life. Except at the end, heart-failure has not been common in this infection. Far more significance lay in the respiration-rate, which rose to much higher figures—fifty-five to sixty—than usually seen in ordinary types of pneumonia, and this before the stages of collapse or cyanosis appeared.

Probably the most reliable sign, and at the same time that of the gravest import, was the onset of this cyanosis. As long as the patient remained definitely red, there was no reason to be more than normally anxious about the case, but as soon as the first tinge of purple developed in the complexion, the prognosis at once became bad. Observers describe a close resemblance between the faces of these men and the cyanosis seen in cases which were gassed in the trenches, but the resemblance is to the eye alone for the influenzal type, although the rate of breathing may be very high, is not accompanied by true respiratory distress.

### Possibilities of Treatment.

So far, active treatment has, for these severe cases, been a more than partial failure, and although the combined work of clinicians and pathologists has resulted in the development of several serum and intravenous methods, yet it seems that the essentials for success are in this, as in the classical type, early recognition and prompt treatment.

American methods described in their journals aim at preparation in all cases which appear to threaten extension to the general system. The pulse being at best a poor indication with an influenzal case, digitalis is given when the temperature is high, and is continued for seventy-two hours, recommenced again, and so on. Taking the patient's weight in pounds and multiplying by two, that number of drops of U.S.A. tinct. digitalis is given in twelve hours, the

**The Pneumonia Mortality—(*continued*).**

heart being thus brought under control to withstand an impending strain. Simultaneously a blood examination is made. A total and differential estimation of the white cells helps to distinguish between the post-influenzal and the croupous lobar pneumonias. It is found that the total count runs abnormally low in the first and correspondingly high in the second type. In the latter the polymorphonuclear cells are much increased, while in the former they may be diminished relatively as well as absolutely.

The Use of Serum.

Taking this indication as a guide, if true lobar pneumonia is expected, the sputum is cultivated in the hope of "typing" the infecting coccus and so placing the case in a definite class, susceptible to a definite stock anti-serum to be at once administered without awaiting the full development of chest signs which in any case may be some days before becoming obvious to clinical examination. If, however, the white count is low, then it is considered that the case is one of the usual mixed infection of influenza, and typing from the sputum is held to be useless.

Use is then made of serum derived from convalescent patients infected during the same epidemic, and most favourable results are claimed. Citrated blood from the same source has been tried, but although theoretically more advantageous, the intravenous injection of whole blood does not appear to be in practice so effective. As before, the injections are commenced at once on the strength of early indications, and not after the patient has definitely developed a severe pneumonia. Mortality figures are certainly in favour of the great utility of these preparative methods. For both stock and autogenous vaccines considerable success is claimed, but these agents are met by much criticism in any except prophylactic use.

Therapeutic Measures.

The value of quinine is now fairly generally recognised both to the influenzal infection and its complications. Intravenous use of the drug has been satisfactorily tried, so also ensol, flavin, and colloidal metals. Antiseptics such as creosote by the mouth, as injections or as inunctions, have been tried. Attempts to localise the general infection with fixation abscesses have been made, and similarly every method of combating microbic disease. For each and all there is claimed some measure of success, and yet in the late epidemics we saw the rapid, apparently unpreventable death of thousands. It was the *severe* type of "influenzal" pneumonia case over which we appear to have no control whatever; others progressed normally.

One has attempted to outline the now widely held view that these cases were in the first place acute septicæmias; in the second they had lesions in their lungs. That also the kidneys, sinuses, ears, meninges were as frequently affected, and that laboratory workers were able to cultivate pathogenic, acutely virulent strepto- and pneumo-cocci from the blood. At such a late stage the infection had all the power in its own hands; the doctor and the patient's body had practically none. Can we not at least impede the systemic extension by preparation and active treatment on organised prophylactic lines immediately after or, in epidemics, before the infection?

This is the attempt in America, and as such worth consideration—not as a new method, but as one which is not generally put into practice.

